# The effect of virtual reality technology on preoperative anxiety in children: a Solomon four-group randomized clinical trial

**DOI:** 10.1186/s13741-019-0116-0

**Published:** 2019-06-04

**Authors:** Fateme Dehghan, Rostam Jalali, Hasan Bashiri

**Affiliations:** 1 Kermanshah University of Medical Sciences, Kermanshah, Iran; 2 Department of Psychology, Faculty of Social Sciences, Razi University, Kermanshah, Iran; 3 Nursing Department, Faculty of Nursing and Midwifery, Kermanshah University of Medical Sciences, Isar Square, Kermanshah, Iran

**Keywords:** Preoperative anxiety, Virtual reality, Children, Medical exposure

## Abstract

**Background:**

Preoperative anxiety correlates positively with the amount of postoperative pain, analgesic use, and length of hospital stay. The purpose of this study was to investigate the effect of virtual reality technology on pre-operative anxiety in children.

**Methods:**

The study utilized Solomon four-group design intervention and a randomized clinical trial. A total of 40 candidates undergoing abdominal surgery were randomly divided into two groups. The pre-operative anxiety scale was assessed by a checklist containing a standardized Yale Preoperative Anxiety Scale questionnaire. The interventional group had a 5-min exposure to the operating room using virtual reality technology, but the control group did not receive virtual reality exposure. The data were analyzed using SPSS-23 software.

**Results:**

Non-parametric test for two independent groups showed no significant difference in baseline scores between interventional 1 and control 1 groups except for vocalization (*p* = 0.019), but there was a significant change in all subscales among intervention group 1 from baseline to post-test (*p* < 0.05) except for state of apparent arousal.

**Conclusions:**

The medical treatment using virtual reality technology, as well as distraction and drowning in the virtual reality, reduced pre-operative anxiety in children.

## Background

Preoperative anxiety is a natural and compatible response to surgery stress which may happen at any time before surgery (Bahrami et al. 2012). This stress is intensified when a patient is informed about the need for surgery, at the initiation of surgery, and during hospitalization (Matthias and Samarasekera 2012; Nourian et al. 2014). Some signs of stress include anxiety, distraction, irritation, fear, and increase in heart rate (Perry et al. 2012). Preoperative anxiety is a potential threat for a patient due to the changes in psychologic responses, including elevation of blood pressure and heart rate, and may endanger patient’s health (Namazi et al. 2014).

Patient anxiety may increase complications of surgery and anesthesia. Preoperative anxiety can reduce the quality of anesthesia and increase demand for analgesics, which can inadvertently prolong the duration of anesthesia. The patient wakes up from anesthesia later, which leads to increase anesthesia and surgery complications. Moreover, anxiety is accompanied by psychologic responses including high blood pressure and cardiac arrhythmia. Generally, there are degrees of preoperative anxiety in all patients, but it is higher in children and requires special attention (Jenkins et al. 2014; Williams and Jones 1968; McCleane and Cooper 1990; Goldman et al. 1988; Jlala et al. 2010). Children, particularly in the early years of their life, are vulnerable to this matter because stress alters the usual pattern of health as well as the safe and familiar environment for the child. On the other hand, children have limited coping mechanisms for resolving stressful situations (Hockenberry and Wilson 2014). Anxiety during hospitalization depends on the evolutionary level of children, history of separation from parents or hospitalization, and an accessible supportive system. Although school children can better accept separation, separation anxiety during hospitalization may increase their need for security and parents. The worst stressful factors in school children during hospitalization are exposure to strange individuals, unfamiliar nutrition pattern, and separation from family, unfamiliar environment, treatment process, and lack of control (Uddin et al. 2002). Preoperative anxiety not only causes pain and discomfort for many children with surgery experience, but also has a negative impact on post-surgical duration of improvement and recovery (Ahmed et al. 2011). Postoperative anxiety behaviors are negative behaviors during surgery experience, including impatience, crying, unintentional urination, and need to physical limiting tools during anesthesia (Wright et al. 2007), and are accompanied by some other postoperative inconsistent behaviors, including distress addition in recovery level, return behavioral disorders after the surgery (nightmare), separation anxiety, eating disorder, and sleep and enuresis disorders (Babashahi et al. 2010).

In fact, some benefits including reducing anxiety are better recovery, quick improvement, reduction of drugs for anesthesia, better pain tolerance, and reduction in hospital stay, which lead to the reduction of costs and complications after the surgery (Babashahi et al. 2010). Anxiety, particularly in children, may cause undesirable mental complications in the future (Bagherian et al. 2012). Anxiety reduction can be performed using two methods including pharmacological and non-pharmacological methods. A non-pharmacological way of reducing anxiety is individual confrontation (Hasani et al. 2009). In recent years, advancements in information and communication technology have led to more effective psychological interventions. Virtual reality and telepsychology are two technologies that are applied in clinical psychology (Botella et al. 2009). Virtual reality can be a potential method for providing public and private healthcare services. It seems that virtual reality can enter psychotherapy process. A panel consisted of 62 psychotherapy specialists tried to respond to the question what is the future of psychotherapy? According to their responses, the usage of only 18 therapeutic interventions out of 38 psychotherapy methods was predicted to increase in the future decade. In this ranking, virtual reality was in the third place (Riva 2005).

North and North observed a feeling of fear in some participants during a military navigation software test. Accordingly, they concluded that this technology not only leads to fear but also can be used for coping with fear and other mental disorders. They introduced virtual reality exposure therapy (VRET) as a new therapeutic method that enables patient exposure to the problems and help them cope with their irrational behaviors. The key issue is that participants, by entering the virtual world, know that there is no danger while they see fearful images similar to real life. This exposure provides a feeling of fear and results in coping (North and North 2016).

The most common application of virtual reality in clinical psychology is in the treatment of phobias. In fact, VRET is introduced as a new tool for therapeutic exposure which is safer, less embarrassing, and cost-effective in comparison with real situations (Rothbaum 2010). The logic behind VRET is simple; patient encounters with deliberate fearful drivers while there is the possibility of reducing patient’s anxiety. Avoiding fearful situations that lead to continuity of fear including situations with frequent encounters (blood sampling and dental extraction procedures) also lead to removing fear (Riva 2005). The VRET is similar to real-world treatment since it causes patients to feel the exposure to motivating drivers of fear. The difference between VRET and traditional behavioral therapy is that graphic image technology, monitors, and input tools simulate an environment the same as real life in laboratory environment. Consequently, VRET provides the feeling of existence for participants with immersion feeling in fearful scene (Riva 2003).

The VRET has advantages over real or imaginary encounter. Firstly, VRET can be executed in usual psychotherapy environments like clinics. Secondly, fearful attributes of a patient can be more effectively separated in comparison with traditional methods (for example, fear for landing in an individual with fear of flying can be repeated in VRET frequently without waiting for a real flight). Thirdly, the immersive nature of VRET can provide a more real experience for motivating emotions than imaginary encounter. Fourthly, this mode can provide effective extinction in fear responses (Riva 2003). Finally, VRET can depict fearful situations with higher intensity than real treatment. Therefore, in cases that real encounter is difficult, VRET can be an alternate for medicinal choices (North and North 2016). Furthermore, patients’ acceptance of virtual reality is high. In the study by Garcia-Palacios et al., the number of sessions of real encounter was compared to VRET. They found that approximately 80% of subjects preferred VRET to real encounter (North and North 2016).

Studies show that patients who use reducing methods for anxiety or compatibility skills before surgery have less anxiety and report lower physical pain after surgery. Furthermore, duration of hospitalization after surgery and patients’ demand for painkillers are reduced in cases who had less preoperative anxiety (Durling et al. 2007). However, there were a few researches which measure the effect of VRET on preoperative anxiety in children. Therefore, the present research considers the effect of VRET on preoperative anxiety in children.

## Methods

The present research was a Solomon four-group interventional study (two experimental groups and two control groups) in randomized controlled trial design. This design is used to remove extraneous effect of pretest on the result of study especially in children.

The participants were children undergoing surgery and gave a written informed consent from legal administrator. Sampling method was convenience (non-random), but allocations to groups were performed randomly by assigning patients with even hospital bed numbers in the interventional group and those with odd hospital bed numbers in the control group. The criteria for inclusion criteria were age between 6 and 12 years old, being a candidate for abdominal surgery, lack of previous history for abdominal surgery, and lack of mental disease, which was performed through studying patient’s medical documents or asking question from their parents/guardians. The exclusion criteria were having stress or special problems in using eyeglass or headphone in VRET. The data collection tool was standardized Yale Preoperative Anxiety Scale questionnaire (Tou et al. 2013). This questionnaire has four domains including activity, vocalization, emotional expressivity, and state of apparent arousal.

Each domain has 4 items that are scored from 1 to 4, which sum to the minimum score of 4 and maximum score of 16.

The sample size was calculated based on the findings of a previous study (Chimeremeze et al. 2013) considering *α* = 5% and *β* = 0/2 using the following equation:

$$ N={\left(1.96+1.28\right)}^2\ {\left(16.3+17.3\right)}^2/{\left(59.1-31.5\right)}^2=8 $$

According to possible sample attrition, the sample size in each group was increased by 20%, which resulted in the addition of 10 patients in each group. The total sample size was therefore 40 children.

**Fig. 1:**
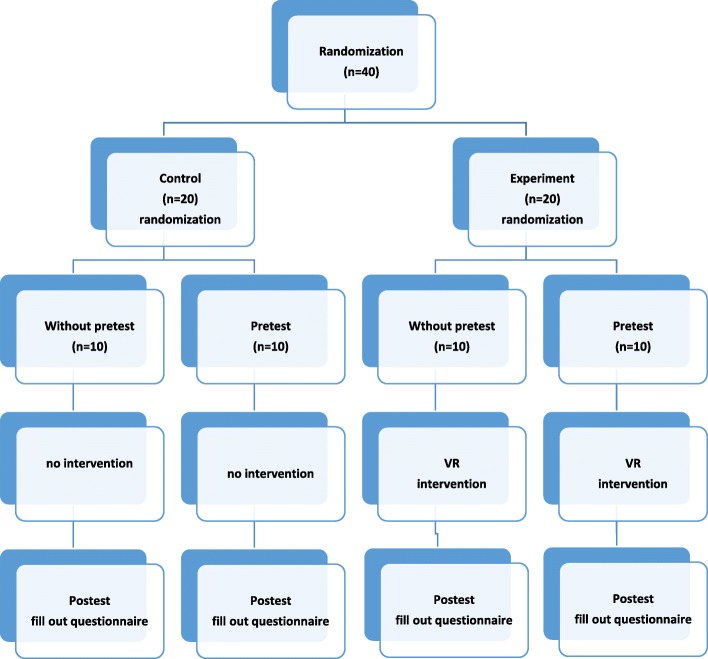
Participants, enrollment, measurement, and intervention

After selecting study samples, participants were divided into two interventional groups and two control groups randomly and the pretest was executed in two groups (interventional 1 and control 1) out of four groups. Subjects in the interventional group encountered the situation through virtual reality by applying eyeglass, which provided a wide field of vision and also provided proper vision for the patient. The patient was placed in front of a computer monitor to present the simulated steps of going to operation room. A headphone was placed on the patient’s ears, and the simulated sounds of entrance to virtual environment were played through the headphones. The intervention was programmed in a way that the virtual drivers could be observed and heard by participants and they were immersed in the virtual environment by involving visual and auditory senses. However, the parents of patients in the control group were requested to touch and caress their children prior to operation. Pretest was performed for one of the interventional and one of the control groups while all groups filled the post-test questionnaire (Fig. 1). The Statistical Package for Social Sciences (SPSS) software version 23 was used for data analysis.

## Results

The study patients included 9 girls (22/5%) and 31 boys (77/5%) with the age of 7/35 ± 2/05 years. Assessment of the normal distribution of data was performed using the Kolmogorov-Smirnov test, which was significant for posttest scores (*p* < 0/05). Therefore, nonparametric tests were used for analysis (Table 1). The Mann-Whitney test showed no statistical difference between groups except for the vocalization domain in pretest (Table 2). For comparing the effect of the intervention, the Kruskal-Wallis (non-parametric test) was used instead of the analysis of variance (ANOVA) and the comparison between groups was performed using the Mann-Whitney test (Table 3).

**Table 1:** One-sample Kolmogorov-Smirnov test

		Activity post	Vocalization post	Emotional expressivity post	State of apparent arousal post
*N*		40	40	40	40
Normal Parameters^a^	Mean	1.90	2.40	2.22	1.68
	Std. deviation	1.236	1.499	1	1.095
Most extreme differences	Absolute	0.342	0.305	0.256	0.406
	Positive	0.342	0.305	0.215	0.406
	Negative	− 0.233	− 0.175	− 0.256	− 0.269
Kolmogorov-Smirnov Z		2.161	1.930	1.618	2.569
Asymp. Sig. (2-tailed)		0.000	0.001	0.011	0.000

^a^Test distribution is normal

**Table 2:** The comparison of post-intervention scores between four groups

Domains	Groups	Mann-Whitney	Kruskal-Wallis
Activity after	E1	E2 *p* = 0.436	*p* = 0.005
		C1 *p* = 0.105	
		C2 *p* = 0.003	
	E2	C1 *p* = 0.393	
		C2 *p* = 0.023	
Vocalization after	E1	E2 *p* = 0.529	*p* = 0.003
		C1 *p* = 0.011	
		C2 *p* = 0.004	
	E2	C1 *p* = 0.105	
		C2 *p* = 0.015	
Emotional expressivity after	E1	E2 *p* = 0.436	*p* = 0.001
		C1 *p* = 0.001	
		C2 *p* = 0.001	
	E2	C1 *p* = 0.035	
		C2 *p* = 0.023	
State of apparent arousal after	E1	E2 *p* = 0.739	*p* = 0.000
		C1 *p* = 0.143	
		C2 *p* = 0.002	
	E2	C1 *p* = 0.218	
		C2 *p* = 0.003	

*E1* experiment 1 (intervention with pretest), *E2* experiment 2 (intervention without pretest), *C1* control 1 (control with pretest), *C2* control 2 (control without pretest)

**Table 3:** The comparison of pre-intervention scores between two groups

Domains	Mann-Whitney		Sig
Activity before	E1	C1	*p* = 0.143
Vocalization before	E1	C1	*p* = 0.019
Emotional expressivity before	E1	C1	*p* = 0.393
State of apparent arousal before	E1	C1	*p* = 0.912

*E1* experiment 1 (intervention with pretest), *C1* control 1 (control with pretest)

The comparison of pre- and post-scores in the interventional groups by Wilcoxon test indicated a significant statistical difference in all domains except for arousals domain. Comparison of pre- and post-scores among control groups showed a significant statistical difference in all domains except for emotional expressivity (Table 4).

**Table 4:** The comparison of pre- and post-intervention scores of each domain

Domains	Wilcoxon		Sig
Activity	E1b	E1a	*p* = 0.026
	C1b	C1a	*p* = 0.334
Vocalization	E1b	E1a	*p* = 0.007
	C1b	C1a	*p* = 0.054
Emotional expressivity	E1b	E1a	*p* = 0.015
	C1b	C1a	*p* = 0.023
State of apparent arousal	E1b	E1a	*p* = 0.063
	C1b	C1a	*p* = 1

*E1* experiment 1 (intervention with pretest), *C1* control 1 (control with pretest), *b* before, *a* after

## Discussion and conclusion

The results of this study showed that children in the interventional groups with pretest-posttests and control groups with posttest had a significant reduction in the preoperative anxiety score after therapeutic exposure using VRET while no significant change in anxiety score of children was observed in the control group. The considerable attraction ability of VRET in distraction from the real world allows the patient to tolerate preoperative anxiety. This finding supports the idea of performing studies to develop distraction techniques. Furthermore, the findings of this study confirm the effect of VRET on unfamiliar and stressful situation exposure like operation room. These findings were in accords with the findings of the studies by Eslami et al. 2015, Afsharian 2016, Memarzadeh et al. 2006, Majzoobi et al. 2013, Robertson et al. 2017, and Shahid et al. 2015.

As it is said, the effects of preoperative anxiety on children are different from postoperative anxiety and are particularly affected by the underlying disease and postoperative improvement, but most individuals believe that preoperative anxiety is determined by private feelings including pressure, impatience, anxiety, and disappointment. In addition, preoperative anxiety, at slight and moderate levels, leads to behavioral improvement after surgery (Nourian et al. 2014), but high preoperative anxiety leads to undesirable psychological and mental consequences (Perry et al. 2012; Namazi et al. 2014; Jenkins et al. 2014).

In children, preoperative anxiety may lead to high mental pressure and negative mental effects, which are accompanied by nightmares and impatience during sleep, separation anxiety, nutritional and growth problems, and fear of medical personnel (Namazi et al. 2014; Jenkins et al. 2014).

These positive results for immersing in VRET support the hypothesis that this media can banish maximum attention from real world and then, allows patients to tolerate preoperative anxiety (Hoffman et al. 2014). In a study, application of VRET reduced preoperative anxiety in children by 30% (Ryu et al. 2017). During cognitive-behavioral treatment for anxiety disorder, patients who were affected by real-life problems and/or through virtual reality in anxiety conditions were compared. Their experience indicated that feeling of presence had a positive relationship with anxiety level. Furthermore, there was a considerable additional effect in stress response, including elevation of serum cortisol level, saliva, and cardiovascular responses, in both real and VRET groups, which indicates the usefulness of VRET programs for the treatment of anxiety (Ling et al. 2014; Kothgassner et al. 2016). Therefore, exposure to VR can be operational and alternative for traditional methods of reducing anxiety because it has further control on anxiety level of the simulator. Children with virtual reality experience have further compatibility during rapid anesthesia than those who received traditional instructions. Children who experienced VRET may be more familiar with the operation room environment and preoperative process than those who experienced usual instruction (Ryu et al. 2018).

Recent studies showed that preoperative expectation can be improved through experience of the new technologies. Furthermore, VRET can be applied for other stressful conditions or similar critical effective factors. Therefore, the findings of the new exciting studies suggest the performance of further researches in this field. Generally, recent studies used functional magnetic resonance imaging (FMRI), which is a neural imaging process which uses MRI technology for the evaluation of brain performance through certain changes in blood flow, suggesting that VRET may reduce psychological responses to a stressful stimulator (pain). Furthermore, mental advantage may explain the reduced mental responses for guiding conscious attention in a recent study. Both heart rate and blood pressure are dynamic attributes that can be generally altered due to response to physical and emotional stimulators. These evaluations are registered concisely in a situation like patient’s movement or exposure to medical personnel (De Queiroz Siqueira et al. 2016).

According to items in movie (360°), the early data of entering the operation room experience for children by application of eyeglass and virtual reality headphone and expectation based on reality lead to cognitive control, which reduces destructive effects of stress (Auerbach et al. 1983). Particularly, the presentation of data in movie format reduces children’s preoperative anxiety and accelerates recovery (Nourian et al. 2014; Tou et al. 2013). Children who find further mental preparation present less signs of emotional disturbance (Afsharian 2016). The observed results can be explained in some psychological theories.

Since children in the interventional group expressed lower anxiety in comparison with the control group, it can be said that VRET to hospital environment, including ward and operation room, provides mental preparation for children and realistic expectation, which are formed in their minds that cause cognitive control, and consequently, the destructive effects of imminent stress are reduced (Jenkins et al. 2014). Moreover, virtual encounter increases the ability for predicting future situations. It is obvious that non-predictable events can cause different kinds of cognitive, emotional, and physical incompatible signs. On the other hand, when children are placed in a naturally painful situation (hospital and expectation for surgery), the probability of anxiety is increased intensely. Prediction ability is an intrinsically desirable matter that can increase the chance for compatibility to stressful factors in children. Therefore, mental processes and sequence, which increase patient’s capacity for predicting stressful events, are desirable and provide the means for improving the compatibility behaviors in children (Memarzadeh et al. 2006).

In cognitive domain, the probability of cooperation is increased when a child watches the movie of his contemporary child, and it is determined for him that his contemporary child also experienced all exposure levels, and he is assured that there is no threat for him in the operation room (Robertson et al. 2017). Considering the improved accessibility to economical devices, the VRET hardware has the potential for widespread use in the society than before (Parsons et al. 2017).

The limitations of this research include lack of consideration of physiological signs of children before surgery (blood pressure and pulse rate), lack of homogenization of the type of surgery, and small sample size. On the other hand, according to the differences in intelligence, social class, level of parental attachment in children, and anxiety of parents, the findings of this study should be cautiously generalized to all children of the same age. In the control group, children received only usual instructions. Statistically, although ANOVA must be used for comparison of groups, due to the non-normally distribution of the data, the alternative non-parametric tests were used.

This study indicated the effectiveness of distraction and immersion in virtual reality for the purpose of encounter exposure on preoperative anxiety in children. Using virtual reality and reducing attention to environment and distraction allow patients to tolerate preoperative anxiety. The VRET virtually exposes the patient to stressful and unfamiliar situations in a safer environment before the real encounter. This technique results in the sensation of familiarity in the real encounter in the patient and thus result in reduced anxiety.

## Data Availability

Datasets can be accessed through the corresponding author upon reasonable request.

## Abbreviations

ANOVA: Analysis of variance
C1: Control 1
C2: Control 2
E1: Experiment 1
E2: Experiment 2
IRCT: Iranian Registry of Clinical Trial
KUMS: Kermanshah University of Medical Sciences
VR: Virtual reality
VRET: Virtual reality exposure therapy
VRET: Virtual reality exposure therapy
